# A cluster‐randomized trial comparing home‐based primary health care and usual clinic care for epilepsy in a resource‐limited country

**DOI:** 10.1002/epi4.12659

**Published:** 2022-10-26

**Authors:** Gagandeep Singh, Suman Sharma, Namita Bansal, Meenakshi Sharma, Anurag Chowdhury, Sarit Sharma, Rajinder K. Bansal, Jatinder S. Goraya, Raj K. Setia, Birinder S. Paul, Josemir W. Sander

**Affiliations:** ^1^ Research & Development Unit Dayanand Medical College Ludhiana India; ^2^ Department of Neurology Dayanand Medical College Ludhiana India; ^3^ UCL Queen Square Institute of Neurology London WC1N 3BG London UK; ^4^ Non‐communicable Diseases Division Indian Council of Medical Research New Delhi India; ^5^ Department of Social & Preventive Medicine Dayanand Medical College Ludhiana India; ^6^ Department of Paediatrics Dayanand Medical College Ludhiana India; ^7^ Punjab Remote Sensing Centre Ludhiana India; ^8^ Chalfont Centre for Epilepsy Chalfont St Peter SL9 0RJ London UK; ^9^ Stichting Epilepsie Instellingen Nederland (SEIN) Heemstede The Netherlands; ^10^ Neurology Department West of China Hospital, Sichuan University Chengdu China

**Keywords:** adherence, low‐ and middle‐income countries, personal impact, secondary treatment gap, seizure control

## Abstract

**Objective:**

To ascertain whether home‐based care with community and primary healthcare workers' support improves adherence to antiseizure medications, seizure control, and quality of life over routine clinic‐based care in community samples of people with epilepsy in a resource‐poor country.

**Methods:**

Participants included consenting individuals with active epilepsy identified in a population survey in impoverished communities. The intervention included antiseizure medication provision, adherence reinforcement and epilepsy self‐ and stigma management guidance provided by a primary health care–equivalent worker. We compared the intervention group to a routine clinic‐based care group in a cluster‐randomized trial lasting 24 months. The primary outcome was antiseizure medication adherence, appraised from monthly pill counts. Seizure outcomes were assessed by monthly seizure aggregates and time to first seizure and impact by the Personal Impact of Epilepsy scale.

**Results:**

Enrolment began on September 25, 2017 and was complete by July 24, 2018. Twenty‐four clusters, each comprising ten people with epilepsy, were randomized to either home‐ or clinic‐care. Home‐care recipients were more likely to have used up their monthly‐dispensed epilepsy medicine stock (regression coefficient: 0.585; 95% confidence intervals, 0.289‐0.881; *P* = 0.001) and had fewer seizures (regression coefficient: −2.060; 95%CI, −3.335 to −0.785; *P* = 0.002). More people from clinic‐care (n = 44; 37%) than home‐care (n = 23; 19%) exited the trial (*P* = 0.003). The time to first seizure, adverse effects and the personal impact of epilepsy were similar in the two arms.

**Significance:**

Home care for epilepsy compared to clinic care in resource‐limited communities improves medication adherence and seizure outcomes and reduces the secondary epilepsy treatment gap.

**BULLET POINTS**

- We sought to determine if home‐care delivered by community workers and primary care workers compared to clinic‐care improves epilepsy outcomes.
- Ours was a community‐based cluster‐randomized trial.
- Participants in home‐care experienced significantly fewer seizures and demonstrated better medication adherence than those in clinic‐care.
- Participants in home‐care were significantly less likely to drop out of care than those in clinic‐care.

## 1 INTRODUCTION

Epilepsy, a common neurological disorder, leads to living with disability and a three‐fold increased risk of premature mortality.^1^, ^2^ It is also associated with several psychiatric and somatic comorbidities, economic losses related to medical care, social exclusion and stigmatisation.^3^, ^4^, ^5^

Low‐ and middle‐income countries (LMICs) are the home to 80% of the world’s people with epilepsy, of whom over two‐thirds remain untreated.^6^ People with epilepsy in these countries probably experience higher risks of premature mortality, morbidity (e.g., injuries) and comorbidities owing to a lack of treatment.^7^, ^8^, ^9^ The use of safe and inexpensive antiseizure medications (ASMs), which results in seizure remission in over 50% of people, could mitigate these risks.^10^, ^11^, ^12^ Several challenges and barriers, however, frustrate the provision of treatment in LMICs.^13^

The WHO encourages primary health care providers to deliver epilepsy care in countries and regions where specialists are few and far apart.^14^, ^15^ Evidence supporting the effectiveness of primary care and community approaches to epilepsy in LMICs is growing, but gaps remain.^13^ One common issue in these programs is the high drop‐out rate. For example, a report from China suggested that over one‐fifth of beneficiaries withdrew from treatment over 2 years for varied reasons. These included a misguided perception of cure in the absence of seizures, frustration over the inability of ASMs to control seizures and adverse effects.^11^ This untimely discontinuation of ASMs by individuals with active epilepsy after treatment initiation is known as the secondary epilepsy treatment gap.^16^

Home delivery of care by trained primary care workers is one suggested approach to reduce the secondary treatment gap. It ensures delivery of ASMs and allows adherence monitoring and reinforcement of self‐management guidance, which might plausibly improve ASM adherence leading to better seizure‐control in individuals with epilepsy. Here, we report the outcome of a community‐based, cluster‐randomized trial of home‐based care provision for epilepsy compared to routine care in a district hospital‐based clinic.^17^

## 2 MATERIAL AND METHODS

The full trial protocol is available elsewhere.^17^ Briefly, the trial took place among poor urban and peri‐urban rural communities in Ludhiana District in Punjab, Northwest India. The district has a population of 1.9 million, served by 1400 registered doctors, with access to six magnetic resonance imaging scanners and about 20 electroencephalographic facilities, all in the private sector. About 80% of the population is literate and 30% are manual labourers. Less than 10% of the population lives in poverty.

A validated questionnaire in the local language was used to screen people for epilepsy.^18^, ^19^ Trained fieldworkers administered it to population clusters of approximately 2000 people divided among nine predetermined immunization sectors in the area (Figure S1). Those who screened positive for epilepsy were invited for evaluation by the study neurologists, 3 T MRI (Skyra, Seimens, Munich, Germany; 20 channels for head coil) brain scan and standard ½‐1 h awake and sleep electroencephalogram (24 channel, Xltek, Ontario, Canada) recordings.

Population‐based screening clusters eventually yielded randomization clusters. The clusters were geographically discrete as shown in Figure S2. The clusters became eligible for randomization and participation once ten individuals with active epilepsy (according to the current International League Against Epilepsy definition^20^) consented in each. Clusters were assigned to either home‐based care or routine clinic‐based care in a 1:1 ratio using a simple computer‐based randomization scheme.^17^ Written informed consent was obtained individually from adults and parents/guardians of children in addition to assent from those of 12–18 years of age before randomization. Sample size calculations adapted to the cluster design, including estimation of intra‐cluster coefficients, are described in the trial protocol.^17^

Participants in the clinic‐based arm attended monthly follow‐up clinics run by a neurologist at the government district hospital. They received routine advice and information and were dispensed with cost‐free ASM/s. Those in the home‐care arm received a monthly visit by care providers together with (i) ASM supply, (ii) guidance about stigma, epilepsy self‐management and first‐aid and social functioning related to schooling, marriage, driving and employment and (iii) medication adherence advice. Care providers were study personnel with qualifications equivalent to auxiliary nurse midwives (ANMs). They delivered the intervention, covering a single cluster in 1 day. Distinct from care providers, a study nurse independently assessed adherence by counting residual pills from the previous month’s dispensing, kept records of seizures and medication adverse effects and applied various questionnaires (see below) in both trial arms. Records of home visits were reviewed at monthly meetings between the study nurse and neurologists, who advised treatment changes if required. The care providers then implemented these during unscheduled home visits. The care providers also facilitated interim visits to the study neurologists among people in the home‐care arm because of failed seizure control, unacceptable side‐effects, and (in women with epilepsy) pregnancy. Similarly, people in the clinic‐based arm could make unscheduled clinic visits or be referred to the study neurologists due to inadequate seizure control, side effects or pregnancy. If they missed follow‐up, they were provided with a week’s stock of ASMs at home and their clinic appointments rescheduled. If they similarly defaulted twice consecutively for reasons other than infirmity or outstation break, they left the trial but were provided ASMs at home if they so desired. ASMs were provided free of charge to all participants.

The primary outcome was ASM adherence, assessed every month by counting residual dosage units of each ASM dispensed during the preceding month. It was considered “acceptable” when the number of dosage units consumed was within ± 2 days of a month’s prescribed use.^11^ When more than 2 days of a month’s use of dosage units remained, adherence was deemed “unacceptable”. When pills were not available (e.g., destroyed, misplaced or lost) for tallying during assessment visits or participants used medication from stocks other than those provided by the trial or when assessments were precluded by locked houses, outstation trips, or during the SARS‐CoV‐19 lockdown (see below), medication adherence was classified as “unknown”. Seizure control was a secondary outcome and evaluated by monthly aggregates of seizures among individuals and clusters, proportion of participants who remained seizure‐free for the trial duration and time to first seizure during follow‐up. Adherence measured on a vernacular version of the self‐reported medication‐taking scale (SRMS) each month, quality of life appraised by a validated vernacular version of the Personal Impact of Epilepsy (PIES) scale, at three‐monthly intervals and attrition determined by drop‐outs in each trial arm were other secondary outcome measures.^21^, ^22^ Outcome assessments were carried out for 24 months instead of 18 months as initially planned following a trial advisory recommendation.

### 2.1 Statistical analysis

Data on socio‐demographic characteristics at baseline, viz current age, gender, ethnic status (native Punjabi Vs interstate immigrant), education, occupation and family income (according to an updated version of the Kuppuswamy scale for socioeconomic status^23^) were collected. Clinical features, including age at onset of epilepsy and seizure frequency in the past year before trial (categorized as daily, weekly, monthly, annual and sporadic), were also noted. Education, occupation and family income were reordered as binary variables. They were compared between the two arms using the Chi‐square test. Seizure frequencies before trial entry were keyed as ordered variables and compared using the Wilcoxon rank‐sum test. Continuous variables were compared using the Student’s *t*‐test.

We analyzed proportions of “acceptable’ adherence and numbers of seizures for each cluster at monthly intervals. Crude cluster‐level measures were compared between the two arms and subsequently adjusted for baseline parameters. We used the z test to compare adjusted cluster‐wise monthly proportions of good adherence in the two arms. Adjusted cluster‐level aggregates of monthly seizures and means of PIES scores were compared using the Student’s *t*‐test.

Data for adherence, numbers of monthly seizures and SRMS and PIES scores were assessed for the patterns of missingness using the missing completely at random t‐test. Missing data points were imputed using the multiple imputations with chained equations technique.^24^ This was in contravention to the planned protocol as the missing‐carry forward approach was deemed impractical for long‐duration drop‐outs.

Four multivariable analyses were performed:

1. A panel‐type random‐effects logistic regression model was fitted with the number of participants with “acceptable” adherence after imputation as the dependent variable and trial arm and month as explanatory variables and baseline characteristics as covariates. The model allowed for between‐cluster variation. The model estimated regression coefficients (with 95% confidence intervals) according to different variables.
2. The numbers of seizures experienced by individuals during each month were fitted after imputation to a panel‐type, negative binomial regression model along with trial arm, month and baseline covariates. The model allowed for between‐cluster variability and included a dummy variable for an interaction between the trail arm and month as seizure aggregates reduced differently with increasing months of follow‐up in the two arms.
3. PIES scores, accrued on a three‐monthly basis, were fitted to a panel‐based linear random‐intercept model with the trial arm as the primary explanatory variable.
4. Attrition, which was different between the two arms, was entered into a binary logistic regression model with the trial arm as the explanatory variable and baseline characteristics as covariates.

Participants also entered a Kaplan‐Meier survival analysis upon randomization, and follow‐up was censored in the event of a seizure, emigration, death or completion of 24 months. The trial arm was the primary independent variable. We used the log‐rank test to compare the seizure‐free survivor function between the two arms.

The Corona virus‐19 pandemic and the ensuing lockdown adversely affected clinic‐ and home‐based services. ASMs were delivered to participants during the lockdown by police courier to ensure continued supply. We performed monthly assessments telephonically and hence, this excluded pill counts. Sensitivity analyses were commissioned to re‐analyze data by excluding reviews during the lockdown period.

We used Stata ver. 15.0 for the analyses. *P* < 0.05 was considered to be significant. The study was scrutinized and approved by the Institutional Ethics Committee of Dayanand Medical College & Hospital, Ludhiana, India (IEC no. 2017–281), and registered with India’s Clinical Trial Registry (Re.: 2017/09/015380). It complied with Consolidated Standards of Reporting Trials (CONSORT), 2010, extension to cluster randomized trials (Table S1). The trial protocol is available at https://researchatdmch.com.

## 3 RESULTS

Screening commenced on 10.07.2017 and was complete by July 23, 2018. The first cluster entered the trial on September 25, 2017. The last cluster was recruited on July 24, 2018. Monthly assessments of the clusters were completed by 01.08.2020. A COVID‐19‐related lockdown was implemented on March 24, 2020, followed by a phased relaxation. It impacted the results as nine of the monthly assessments (2 assessments of 3 clusters each and one in another cluster in home‐care and two assessments of one cluster in clinic‐care) took place during this period. The distributions of seizure frequency at baseline and other characteristics were similar in the two trial arms (Table 1).

**TABLE 1:** Comparison of baseline socio‐demographic and clinical variables in the home‐care and clinic‐care arms of the trial

Characteristic	Category	Clinic‐based arm (n = 120)	Home‐based arm (n = 120)	Statistical significance (P value)
Age (years)				0.351^#^
Mean ± SD		26 ± 15	27 ± 15	
95% Confidence Intervals		23–28	24–30	
Median (IQR)		23 (15–33)	26 (15–38)	
Age of onset of epilepsy				0.690^#^
Mean ± SD		15 ± 14	14 ± 13	
95% Confidence Intervals		12–17	12–16	
Median(IQR)		12 (5–19.75)	12(3–20)	
Duration of epilepsy (years)				0.244^#^
Mean ± SD		13 ± 10	15 ± 12	
Median(IQR)		10 (4–20)	12 (6–21)	
95% Confidence Interval		11–15	13–17	
Gender	Female	39 (33%)	40 (33%)	0.891^##^
Religion	Hindu	74 (62%)	65 (54%)	0.324^##^
	Sikh	44 (37%)	50 (42%)	
	Others	2 (2%)	5 (4%)	
Ethnic origin	Local	73 (61%)	77 (64%)	0.594^##^
	Interstate migrant	47 (39%)	43 (36%)	
Education**	High school and below	109 (91%)	104 (87%)	0.307
	Post‐High school	11 (9%)	16 (13%)	
Occupation**	Employed	42 (35%)	46 (38%)	0.592^##^
	Unemployed	78 (65%)	74 (62%)	
Income**	<INR 18000/month	115 (96%)	115 (96%)	1.000^##^
	>INR 18000/month	5 (4%)	5(4%)	
Social Class**	Lower	98 (82%)	96 (80%)	0.743^##^
	Upper	22 (18%)	24 (20%)	
Marital Status	Married	42 (35%)	47 (39%)	0.504^##^
	Single/Divorced/Separated	78 (65%)	73 (61%)	
Habitat	Urban	106 (88%)	97 (81%)	0.108^##^
	Rural	14 (12%)	23 (19%)	
Pre‐trial seizure frequency***	Daily	10 (8%)	9 (8%)	0.150^###^
	Weekly	11 (9%)	13 (11%)	
	Monthly	41 (34%)	32 (27%)	
	Annual	9 (8%)	7 (6%)	
	Biannual	19 (16%)	15 (13%)	
	Sporadic	30 (25%)	44 (37%)	

*statistically significant; **modified according to Kuppuswamy scale for socioeconomic status, version 2015 (Ref. 19); *** in past 2 years; ^#^Mann–Whitney test (as data not normally distributed), ^##^Chi‐square test, ^###^ Wilcoxon rank test.

Abbreviations: INR, Indian National Rupees; IQR, interquartile range; SD, standard deviation.

Pill count‐based adherence outcomes were missing in 1050 (18.2%), seizure counts in 764 (13.3%) and SRMS scores in 1137 (19.7%) of 5760 data points each and PIES scores in 204 (10.6%) of 1920 data points. All variables were found to be missing at random.

By trial‐end, more people in‐clinic‐care (n = 44; 37%) than in‐home‐care (n = 23; 19%) exited the trial (*P* = 0.003). Four participants died in each arm during the trial. The remaining exited due to emigration (n = 15; 4 in‐home‐care), failed clinic attendance (n = 12; all in clinic‐care), completion of treatment due to remission or withdrawal due to adverse effects (n = 14; 9 in‐home‐care) or failure to control seizures (n = 3; one in home‐care), and other reasons (n = 15; 5 in‐home‐care). Those who failed clinic attendance or opted to withdraw because of dissatisfaction were still provided with ASMs. Hence, seizure control could be assessed by phone or otherwise in 89 (74%) participants in the clinic care arm and 105 (87.5%) in‐home care arm. Baseline characteristics of drop‐outs and those who completed the trial were similar for adherence assessments and seizure control (Tables S2a,b and S3). We examined all clusters in each arm for the primary and secondary outcomes.

Home care recipients were more likely to be adherent than those in clinic‐based care (regression coefficient: 0.585; 95% confidence intervals, 0.289 to 0.881; *P* = 0.0001) in the imputed, panel‐type cluster‐averaged, random effects logistic regression model based on adherence categories (Table 2). This meant that they were 1.79 more likely to have used their monthly ASM stocks in comparison to those in clinic care.

**TABLE 2:** Multivariate model for pill‐count based antiseizure medication adherence and monthly seizure aggregates

Variables (reference category)	ASM adherence:Regression coefficients); *P*‐value	Monthly seizure aggregates: Regression coefficients (95% CI); *P*‐value
Home‐care arm	0.585 (0.289 to 0.881); *P* = 0.0001*	‐2.060 (−3.335 to −0.785) *P* = 0.002
Assessment month	0.039 (0.017 to 0.062); *P* = 0.001	−0.417 (−0.52 to −0.32) *P* = 0.0001
Age at entry to trial	−0.002 (−0.013 to 0.009); *P* = 0.64	0.001 (−0.018 to 0.020) *P* = 0.89
Age of onset of epilepsy	−0.0003 (−0.019 to 0.019); *P* = 0.0.98	−0.019 (−0.041 to 0.003) *P* = 0.09
Gender (female)	−0.057 (−0.377 to 0.261); *P* = 0.72	0.114 (−0.361 to 0.589) *P* = 0.64
Ethnic origin (Migrant)	0.181 (−0.071 to 0.433) *P* = 0.16	0.262 (−0.76 to 0.600) *P* = 0.13
Education (High school and above)	0.027 (−0.329 to 0.382); *P* = 0.88	0.232 (−0.195 to 0.658) *P* = 0.29
Occupation (Employed)	0.147 (−0.225 to 0.518); *P* = 0.44	−0.164 (−0.698 to 0.369) *P* = 0.55
Family Income (< INR 18000/month)	0.081 (−0.207 to 0.368); *P* = 0.58	0.072 (−0.258 to 0.403) *P* = 0.67
Pre‐trial seizure frequency (Daily)		
Weekly seizures	0.287 (−0.345 to 0.919); *P* = 0.37	0.655 (−0.054 to 1.363) *P* = 0.07
Monthly seizures	0.447 (−0.069 to 0.962); *P* = 0.09	−0.187 (−0.733 to 0.358) *P* = 0.50
Biannual seizures	0.447 (−0.252 to 1.146); *P* = 0.21	−1.332 (−2.054 to −0.609) *P* = 0.0001
Annual seizures	0.410 (−0.247 to 1.067); *P* = 0.22	−1.159 (−2.357 to 0.0398) *P* = 0.058
Sporadic seizures	0.639 (0.041 to 1.239); *P* = 0.04	−1.304 (−1.990 to −0.617) *P* = 0.0001

*Note*: Number of observations = 4996 (Average observations per group = 21.1; range (1–24); Overall probability > chi2 = 0.00001 for adherence model; Overall probability > chi2 = 0.00001 in the negative binomial regression model for monthly seizure aggregates; Log likelihood = −3720.422* ‐ Statistically significant; Parentheses in columns 1 and 2 (variables) contain the reference category against which comparison was made.

Regression coefficients for acceptable vs. unacceptable or unknown ASM adherence (based on pill counts) spread over 24 months after imputation in the random effects logistic regression model (column 2) and for monthly seizures in a cluster‐averaged panel‐type negative binomial regression model (column 3) are presented

Individuals and clusters in home‐care had more seizures than those in‐clinic care in the early months of the trial but overall had fewer seizures across the entire trial span (regression coefficient: −2.060; 95%CI, −3.335 to −0.785; *P* = 0.002 in the panel‐type negative binomial regression), particularly during its last segment (Tables 2 and 3; Figure 2A,B. During an observation period amounting to 71 541 days, 142 participants, 72 (60%; against expected failures of 66) in clinic‐care and 70 (58%; against an expected number of 77) in home‐care, had at least one seizure. Both arms had similar survivor function (*P* = 0.27) (Figure 3).

**TABLE 3:** Comparison of monthly cluster‐wise seizure aggregates and their effect estimates in the two arms of the trial

Month	Unadjusted means of monthly seizure aggregates		Adjusted means of monthly seizure aggregates*		Effect estimate					
	Clinic‐ based	Home‐ based	Clinic ‐based	Home‐ based	Unadjusted			Adjusted		
	Mean ± SD (95% CI)	Mean ± SD (95% CI)	Mean ± SD (95% CI)	Mean ± SD (95% CI)	Difference**	Ratio^#^	*P*‐value	Difference**	Ratio^#^	*P*‐value
1	11.5 ± 18.74 (−0.41 to 23.41)	16.75 ± 26.28 (0. 05 to 33.45)	15.55 ± 16.87 (4.83 to 26.27)	12.95 ± 12.54 (4.99 to 20.92)	5.25	1.46	0.579	−2.60	0.83	0.673
2	8.50 ± 13.74 (−0.23 to 17.23)	10.92 ± 22.17 (−3.17 to 25.01)	13.12 ± 17.6 (1.94 to 24.30)	8.79 ± 10.41 (2.18 to 15.40)	2.42	1.28	0.751	4.33	0.67	0.471
3	10.83 ± 17.17 (−0.07 to 21.74)	10.92 ± 17.37 (−0.11 to 21.95)	12.69 ± 16.62 (2.12 to 23.25)	10.83 ± 12.24 (3.05 to 18.61)	0.09	1.01	0.991	1.86	0.85	0.758
4	13.75 ± 20.21 (0.91 to 26.59)	8.33 ± 13.64 (−0.33 to 17.0)	14.26 ± 16.23 (3.95 to 24.57)	10.22 ± 11.08 (3.18 to 17.26)	−5.42	0.61	0.450	4.04	0.72	0.484
5	11.33 ± 17.41 (0.27 to 22.39)	11.08 ± 14.78 (1.69 to 20.47)	16.42 ± 16.29 (6.07 to 26.77)	12.01 ± 10.19 (5.54 to 18.48)	−0.25	0.98	0.970	4.41	0.73	0.435
6	9.25 ± 14.97 (−0.26 to 18.76)	8.58 ± 12.89 (0.39 to 16.78)	10.46 ± 8.41 (5.12 to 15.81)	9.03 ± 7.96 (4.25 to 14.36)	−0.67	0.93	0.908	1.43	0.86	0.731
7	7.92 ± 14.90 (−1.55 to 17.39)	8.5 ± 10.99 (1.52 to 15.48)	11.16 ± 9.69 (5.0 to 17.31)	9.03 ± 7.17 (4.48 to 13.59)	0.58	1.07	0.914	−7.87	7.78	0.548
8	10.17 ± 15.87 (0.08 to 20.25)	7.42 ± 7.53 (2.64 to 12.20)	15.04 ± 13.28 (6.61 to 23.48)	9.83 ± 6.23 (5.88 to 13.78)	−2.75	0.73	0.593	5.21	0.65	0.231
9	7.17 ± 11.54 (0–0.16 to 14.50)	4.00 ± 5.49 (0.51 to 7.49)	10.96 ± 9.86 (4.70 to 17.23)	6.94 ± 6.30 (2.94 to 10.95)	−3.17	0.56	0.400	4.02	0.63	0.247
10	8.17 ± 12.18 (0.43 to 15.91)	4.17 ± 5.24 (0.84 to 7.49)	15.28 ± 20.18 (2.46 to 28.10)	7.29 ± 6.69 (3.04 to 11.53)	−4.00	0.51	0.307	7.99	0.48	0.206
11	8.75 ± 13.86 (−0.05 to 17.55)	6.17 ± 7.85 (1.18 to 11.15)	10.36 ± 8.48 (4.98 to 15.75)	7.62 ± 5.05 (4.42 to 10.83)	−2.58	0.71	0.580	2.74	0.74	0.347
12	5.92 ± 12.29 (−1.89 to 13.72)	3.17 ± 3.71 (0.81 to 5.53)	4.93 ± 3.64 (2.62 to 7.25)	4.49 ± 3.39 (2.34 to 6.65)	−2.75	0.54	0.466	0.44	0.91	0.762
13	8.00 ± 16.31 (−2.36 to 18.36)	3.25 ± 3.74 (0.87 to 5.63)	5.59 ± 5.45 (2.13 to 9.05)	5.80 ± 7.3 (1.16 to 10.45)	−4.75	0.41	0.336	−0.21	1.04	0.939
14	12.42 ± 22.21 (−1.70 to 26.53)	3.67 ± 3.8 (1.25 to 6.08)	100.27 ± 163.97 (−3.92 to 204.45)	29.32 ± 27.33 (11.95 to 46.68)	−8.75	0.30	0.192	70.95	0.29	0.153
15	8.08 ± 15.93 (−2.04 to 18.21)	4.92 ± 5.79 (1.24 to 8.60)	5.53 ± 5.71 (1.91 to 9.16)	7.49 ± 8.4 (2.15 to 12.83)	−3.16	0.61	0.524	−1.96	1.35	0.512
16	9.17 ± 18.75 (−2.74 to 21.07)	4.50 ± 5.09 (1.27 to 7.73)	6.77 ± 7.15 (2.23 to 11.31)	6.91 ± 8.11 (1.76 to 12.07)	−4.67	0.49	0.414	−0.14	1.02	0.964
17	7.08 ± 13.1 (−1.24 to 15.41)	4.67 ± 6.64 (0.45 to 8.89)	5.27 ± 6.23 (1.31 to 9.22)	6.48 ± 8.61 (1.02 to 11.96)	−2.41	0.66	0.574	−1.21	1.23	0.694
18	6.33 ± 12.86 (−1.84 to 14.50)	3.17 ± 3.93 (0.67 to 5.66)	4.23 ± 4.99 (1.06 to 7.41)	5.3 ± 6.61 (1.10 to 9.49)	−0.16	0.97	0.423	−1.07	1.25	0.661
19	6.33 ± 11.6 (−1.04 to 13.71)	4.17 ± 6.00 (0.37 to 8.0)	5.04 ± 6.49 (0.92 to 9.17)	5.48 ± 8.44 (0.12 to 10.84)	−2.16	0.66	0.571	−0.44	1.09	0.888
20	0.96 ± 4.65 (−0.03 to 1.94)	0.71 ± 3.07 (0.12 to 1.30)			−0.25	0.74	0.658			
21	0.88 ± 4.56 (−0.08 to 1.84)	0.60 ± 3.41 (−0.06 to 1.26)	0.75 ± 2.32 (0.33 to 1.17)	0.67 ± 3.16 (0.10 to 1.24)	−0.28	0.68	0.630	0.08	1.12	0.838
22	6.00 ± 11.58 (−1.36 to 13.36)	2.50 ± 2.58 (0.86 to 4.14)	5.68 ± 5.84 (1.97 to 9.39)	4.77 ± 6.64 (0.55 to 8.98)	−3.50	0.42	0.318	0.91	1.19	0.723
23	5.67 ± 11.4 (−1.58 to 12.91)	2.17 ± 2.44 (0.61 to 3.72)	5.4 ± 5.75 (1.74 to 9.05)	4.1 ± 6.69 (−0.15 to 8.35)	−3.50	0.38	0.310	1.30	1.32	0.616
24	5.67 ± 11.43 (−1.59 to 12.93)	1.83 ± 1.95 (0.60 to 3.07)	5.1 ± 5.56 (1.57 to 8.64)	3.97 ± 6.46 (−0.14 to 8.07)	−3.84	0.32	0.264	1.13	1.28	0.648

*Note*: *Adjusted for baseline demographic and clinical variables as well as duration of participation in the trial; ** Effect estimate, i.e., difference in monthly means of seizure aggregates between the two arms, adjusted for baseline demographic and clinical variables as well as duration of participation in the trial = Mean of seizure aggregates in home‐care – mean of seizure aggregates in clinic ‐care; # ratio of means of seizure aggregates between the two arms, adjusted for baseline demographic and clinical variables as well as duration of participation in the trial = Mean of seizure aggregates in home‐care/mean of seizure aggregates in clinic‐care. The above analysis is presented without imputation.

Crude and adjusted effect estimates derived from cluster‐level analyses of seizures aggregates, the proportions of participants with “acceptable” adherence at monthly intervals, and SRMS and PIES scores in the two arms are shown in Tables 3 and S4‐S6.

### 3.1 Ancillary analyses

During the observation period, people in home‐care undertook 326 interim visits to the clinic (amounting to 1.48 ± 0.46 [standard deviations; SD] visits/person‐year) on the advice of and facilitation by care providers. Those in clinic‐based care made 57 unscheduled visits and were referred to higher‐level care on 35 occasions. There were 20 unplanned or emergency visits to the hospital by home‐care recipients compared to 47 in clinic‐based care. Participants in‐home care were hospitalized on 25 occasions compared to 26 times in clinic‐care. Treatment changes, including dose and medications, were implemented on 224 occasions in home‐care compared to 165 times in clinic‐based care. ASM/s were discontinued in five people in‐home care and two in clinic‐care. Two in‐home care had seizure recurrence following the discontinuation. The numbers of spontaneous adverse effects captured during monthly assessments were similar in the two arms (Table S7; incidence rate ratio: 0.94; 95%CI, 0.77‐1.15; *P* = 0.1).

### 3.2 Sensitivity analyses

We repeated the analyses by excluding assessments during the lockdown period. Pill count‐based adherence was found similar in the two arms in a random‐effects logistic regression model restricted to the first 18 months (Table S8; regression coefficient: 0.17; 95%CI, −0.29‐0.63; *P* = 0.74). There were significantly fewer seizures in the home‐care arm (Table S9; regression coefficient: ‐2.14; 95%CI, −3.14 to −0.84; *P* = 0.001) in the first 18 months.

## 4 DISCUSSION

**FIGURE 1:**
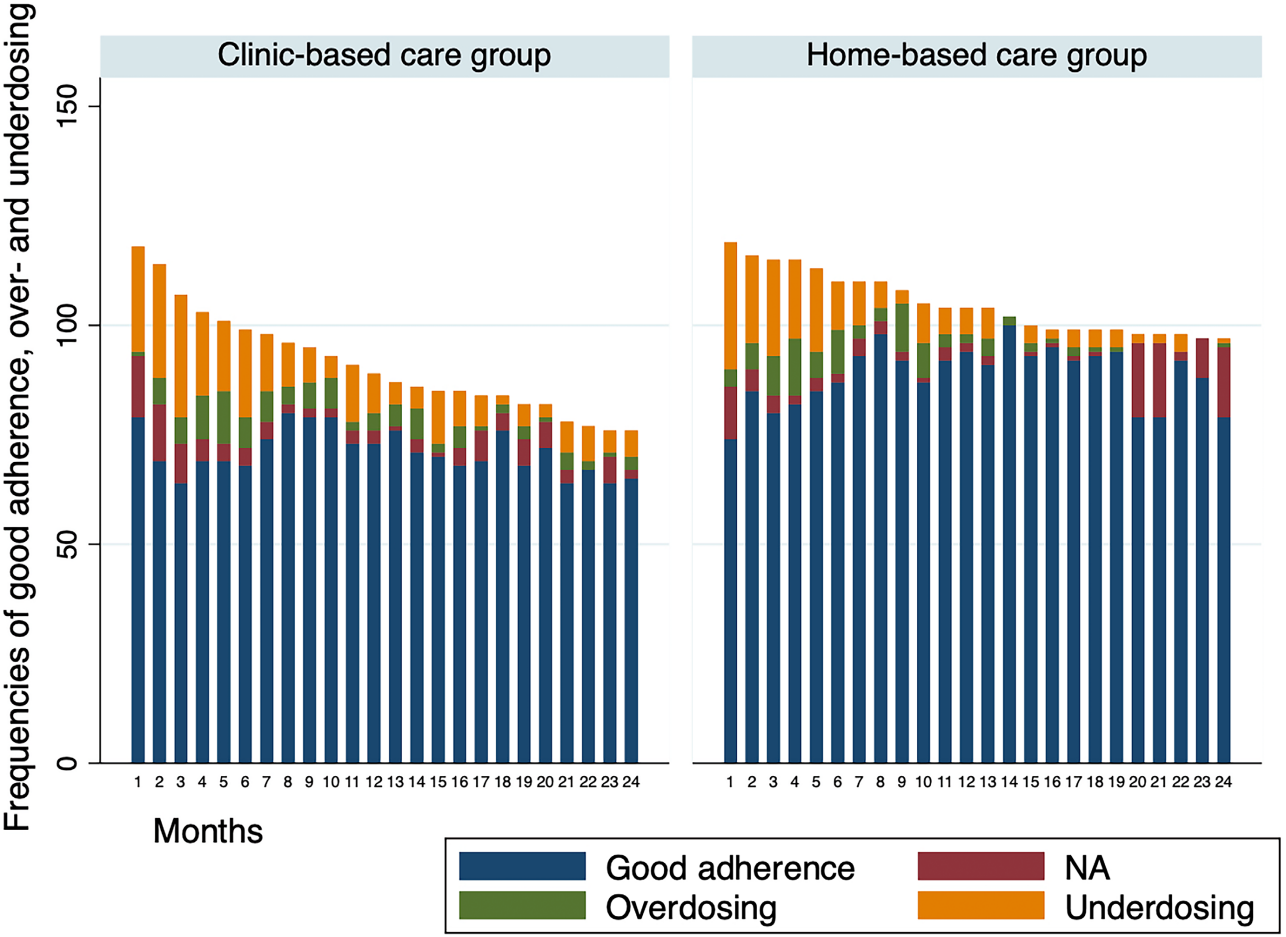
Monthly medication adherence measured by pill counts in the two arms

**FIGURE 2:**
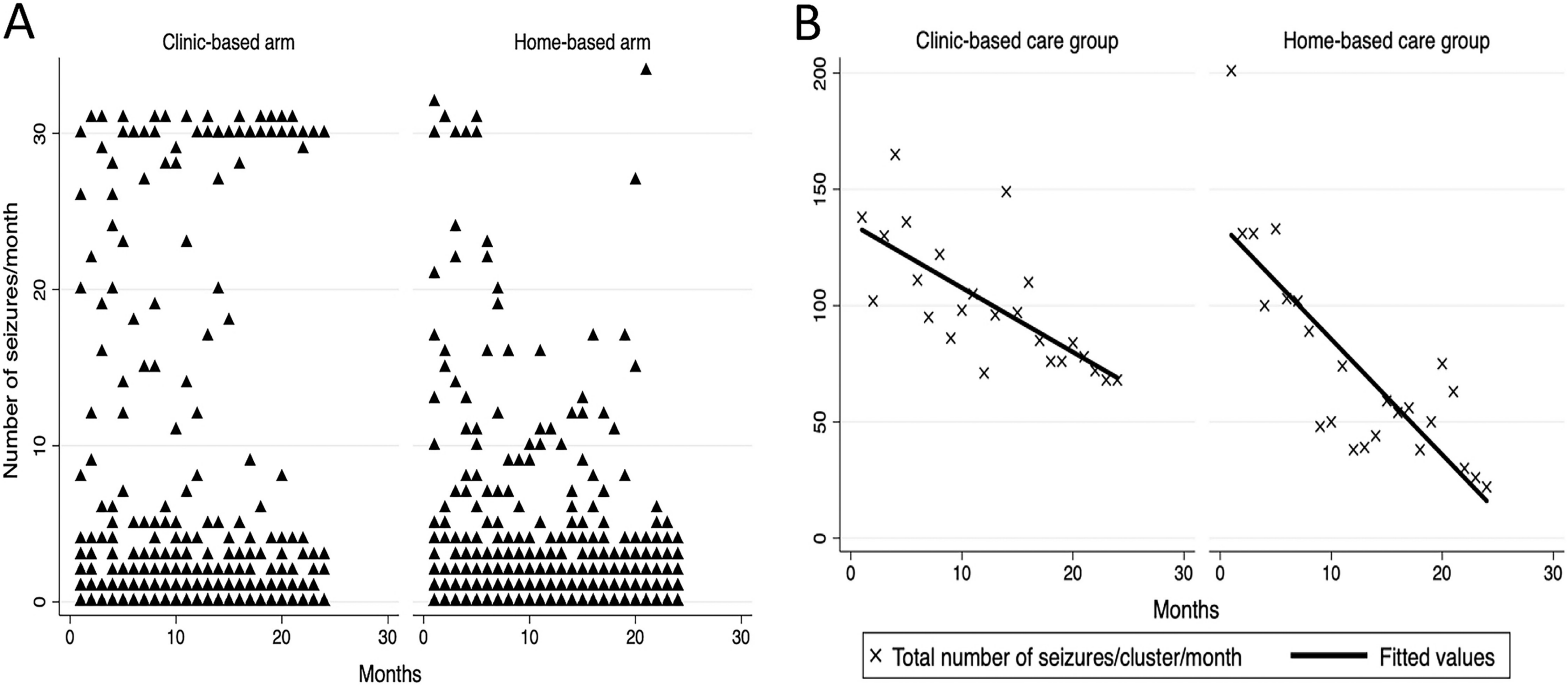
Monthly seizure aggregates in individual subjects (A) and in clusters (B) in the two arms

Home‐care recipients had better medication adherence as measured by monthly pill counts and fewer seizures than those attending the clinic (Tables 2, 3; Figure 1). They had more seizures than those in the clinic care arm in the early months of the trial, but they eventually experienced fewer seizures as the trial progressed (Figures 2A,B). Other seizure outcomes measured by stipulated criteria were similar in the two arms. Notably, participants in‐home care were significantly less likely to drop out than those attending the clinic.

**FIGURE 3:**
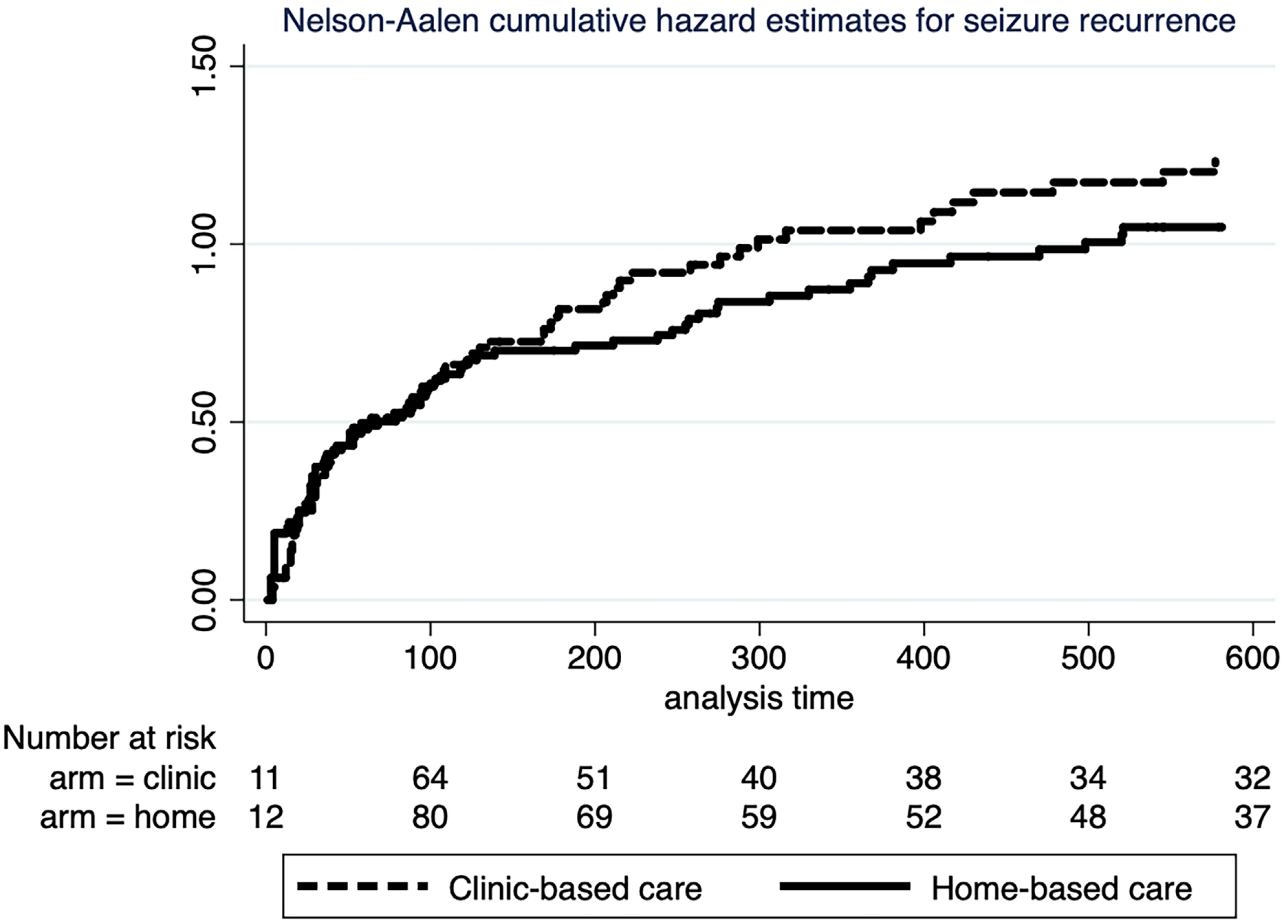
Nelson‐Aalen cumulative hazard curves for first seizure recurrence in the two arms

The choice of the study area was fairly representative of urban poverty‐stricken communities in LMICs. A randomized cluster design was employed to ensure representation across the study area and prevent contamination by the intervention. ASM adherence was the primary outcome as it was an immediate downstream effect of the intervention. We used a stringent criterion for acceptable adherence as used in a successful demonstration project in China and because missing even a single dose can lead to seizures in epilepsy.^11^ We hypothesized that home‐care would lead to better seizure outcomes by improving adherence through motivational campaigns and home provision of ASMs.

Apart from the findings, we share some of the challenges faced in conducting a population‐based trial in resource‐constrained communities with little disease and treatment literacy. The greatest challenge was the Corona virus‐19‐related lockdown towards the trial’s end. We overcame the restrictions by ensuring ASM distribution to all participants during this period. The lockdown, however, precluded monthly face‐to‐face assessments by the nurse, and phone reviews substituted these. All outcomes were appraised during these telephonic assessments apart from pill counts, which required physical tallying monthly residual dosage units. Four clusters in the home‐care arm were assessed during the lockdown period instead of one in clinic‐based care. As clusters were randomized soon after their assembly, they commenced and completed the trial at different time points. Thus, pill counts were indeterminate more often for the home‐care group.

Low literacy and a lack of understanding among the trial participants was another challenge, which was perhaps reflected in the inability of trial questionnaires, i.e., the SRMS and PIES, to capture the intended outcomes and bring out any differences between the two arms (Tables 1, S5 and S6). Dealing with stigma and disease and treatment‐related perceptions of impoverished communities with low literacy levels was apparent, especially during the recruitment and trial start.^25^ Besides, sensitisation towards epilepsy among primary healthcare providers was lacking, which was overcome by continuing epilepsy education.^26^

The lack of stringent blinding, implicit with a community‐based trial, constrains the interpretation of the results. The disparate assemblies of covariates might also have impacted the outcomes. Some of the findings might be subject to Type 2 errors. Besides, although we recruited subjects after a door‐to‐door campaign, we did not randomize the subjects immediately after identification and ascertainment. As a result, this trial does not cover case identification and ascertainment. Finally, the inherent disadvantage of using pill counts as a surrogate for medication adherence is acknowledged. It is impossible to ensure whether the pills were taken or simply thrown away.

Despite all limitations, the higher attrition in the clinic‐based arm is a robust argument favoring home care for epilepsy. It was likely underpinned by poverty as most trial participants were impoverished, often daily‐wagers, having to cope with travel expenses and loss of wages to make clinic visits. Home care offsets the financial losses incurred on clinic visits. Studies from other regions, e.g., China, Kenya and Zimbabwe, have shown that clinic attendance declines with time, often explained by erratic ASM supplies, considerable distance (from clinics), poor treatment literacy, and specifically lack of knowledge about the duration of treatment.^11^, ^27^, ^28^, ^29^

Home‐care has been applied to improve outcomes in chronic medical disorders, notably hypertension, with mixed results.^30^ It has also been implemented for epilepsy previously, e.g., in Kenya, Laos and Cambodia.^12^, ^31^, ^32^, ^33^ The studies from communities in Laos and Cambodia used facility‐ and community‐based health visitors. Our model encompassed home‐care provided by the community‐ and primary care providers with backup by neurologists. The model might be feasible in India with a fair availability of neurologists but might be a limiting factor in other low‐income countries due to the scarcity of neurologists. The availability of tools to investigate epilepsy, e.g., electroencephalography and MRI might also be constrained in low‐income countries.

Regardless of the means and modalities of implementation, home‐based care for epilepsies appears promising in underserved communities in LMICs. The substantial reduction in the secondary epilepsy treatment gap is a strong argument favoring home‐care for epilepsy. In addition, it can provide better outcomes in terms of medication adherence and seizure control. Eventually, the success of any home‐care model will depend on how it addresses barriers to care‐seeking behaviors and by ensuring a continued supply of ASMs and cost‐effectiveness. Task‐sharing between primary health care providers and specialists is a credible option and should be rigorously tested in the field environment in implementation trials in LMICs.

## AUTHOR CONTRIBUTIONS

Gagandeep Singh; (1) conceptualized and designed the study; (2) drafted the manuscript; (3) performed neurological evaluations; (4) supervised treatment plans; (5) is the study guarantor. Suman Sharma; (1) undertook field data collection; (2) assisted in data analysis; (3) reviewed the manuscript. Namita Bansal; (1) Performed the statistical analysis; (2) reviewed the manuscript. Meenakshi Sharma; (1) provided a substantial intellectual contribution to the study design; (2) reviewed the manuscript. Anurag Chaudhary; (1) provided a substantial intellectual contribution to the study design; (2) supervised the field study; (3) reviewed the manuscript. Sarit Sharma; (1) provided a substantial intellectual contribution to the study design; (2) supervised the field study; (3) was the randomization officer (4) reviewed the manuscript. Rajinder Bansal; (1) provided a substantial intellectual contribution to the study design; (2) supervised record and data‐keeping; (3) performed neurological evaluations; (4) reviewed the manuscript. Jatinder S. Goraya; (1) provided a substantial intellectual contribution to the study design; (2) supervised record and data‐keeping; (3) performed neurological evaluations; (4) reviewed the manuscript. Raj K Setia; (1) provided a substantial intellectual contribution to the study design; (2) performed the geographic information mapping for the field operations; (3) reviewed the manuscript. Birinder S Paul; (1) provided a substantial intellectual contribution to the study design; (2) supervised record and data‐keeping; (3) performed neurological evaluations; (4) reviewed the manuscript. Josemir W. Sander; (1) provided critical intellectual input to the study’s conceptualisation, design, analysis and revision of the manuscript. All reviewed and approved the submitted manuscript.

## FUNDING INFORMATION

The study was funded by an Ad hoc grant no. No:5/4–5/127/Neuro/2013‐NCD‐I of the Indian Council of Medical Research.

## CONFLICT OF INTEREST

Gagandeep Singh; received personal support from Novartis, Sanofi and Abbott India outside of the submitted research. Suman Sharma; received salary support from the Indian Council of Medical Research. Namita Bansal, Meenakshi Sharma, Anurag Chaudhary, Sarit Sharma, Rajinder Bansal, Jatinder S. Goraya, Raj K Setia, and Birinder S Paul has no conflict of interest to declare. Josemir W. Sander; reports personal fees from Eisai, UCB, Arvelle and Zogenix, grants from Eisai, UCB and GW Pharma, outside the submitted work.

## DATA AVIALABILITY STATEMENT

We will grant access to independent investigators to anonymised individual subject data, including trial database, protocols, CRFs, and reports 12 months after publication of this paper. Before use, the researcher/s need to apply to the Dayanand Medical College & Hospital Institutional Review Board using an application form available at https://dmch.edu/specialists_hospital.php?Id=NTM=. Following IRB approval, a formal data sharing agreement needs to be signed.

## ETHICAL APPROVAL

We confirm that we have read the Journal’s position on issues involved in ethical publication and affirm that this report is consistent with those guidelines.

## Supporting information


Figure S1

Figure S2

Appendix S1
