# Supplementary figures and images for: A cluster‐randomized trial comparing home‐based primary health care and usual clinic care for epilepsy in a resource‐limited country
Source: Epilepsia Open. 2022 Oct 26;7(4):781–91. doi: 10.1002/epi4.12659 (PMC9712458; doi:10.1002/epi4.12659)

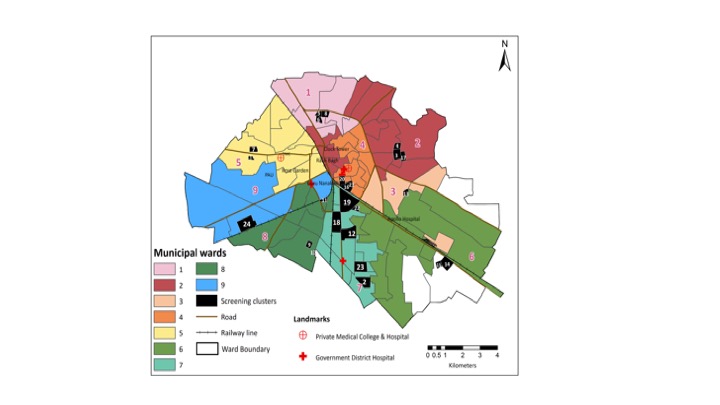

Supplement: Supplementary file 1 — Figure S1 [file EPI4-7-781-s001.jpg]

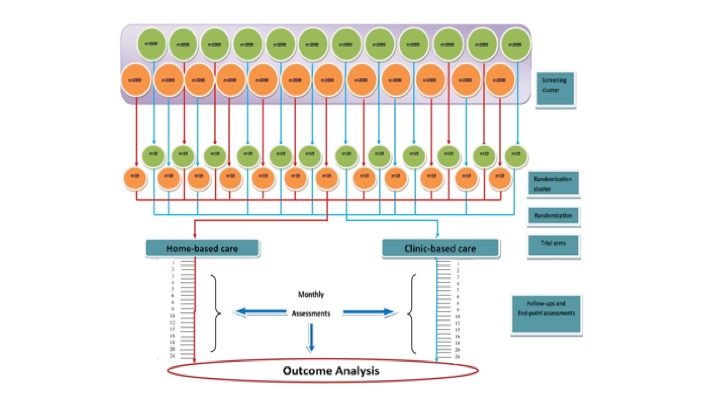

Supplement: Supplementary file 2 — Figure S2 [file EPI4-7-781-s002.jpg]
