# Supplementary material for: A cluster‐randomized trial comparing home‐based primary health care and usual clinic care for epilepsy in a resource‐limited country
Source: Epilepsia Open. 2022 Oct 26;7(4):781–91. doi: 10.1002/epi4.12659 (PMC9712458; doi:10.1002/epi4.12659)
Supplement: Supplementary file 3 — Appendix S1 [file EPI4-7-781-s003.docx]

**Home-based care for people with epilepsy: a community-based, cluster-randomized trial using primary care resources.**

Supplementary Tables

Table s1: **CONSORT 2010 checklist of information to include when reporting a cluster randomised trial**

| Section/Topic | Item No | Standard Checklist item | Extension for cluster designs | Page No * |
| --- | --- | --- | --- | --- |
| Title and abstract | | | |  |
|  | 1a | Identification as a randomised trial in the title | Identification as a cluster randomised trial in the title | Yes; Page 1 |
|  | 1b | Structured summary of trial design, methods, results, and conclusions (for specific guidance see CONSORT for abstracts)^[[1]](#endnote-1),^^[[2]](#endnote-2)^ | See table 2 | Yes; Pages 3 & 4 |
| Introduction | | | |  |
| Background and objectives | 2a | Scientific background and explanation of rationale | Rationale for using a cluster design | Yes, Page 6; Lines 111-113 |
|  | 2b | Specific objectives or hypotheses | Whether objectives pertain to the the cluster level, the individual participant level or both | Yes, Page 6, Lines 106-107 |
| Methods | | | |  |
| Trial design | 3a | Description of trial design (such as parallel, factorial) including allocation ratio | Definition of cluster and description of how the design features apply to the clusters | Yes, Page 7, Lines 129-136 |
|  | 3b | Important changes to methods after trial commencement (such as eligibility criteria), with reasons |  | NA |
| Participants | 4a | Eligibility criteria for participants | Eligibility criteria for clusters | Yes, Page 7, Lines 129-132 |
|  | 4b | Settings and locations where the data were collected |  | Page 6, Lines 117-122; Fig. 1s |
| Interventions | 5 | The interventions for each group with sufficient details to allow replication, including how and when they were actually administered | Whether interventions pertain to the cluster level, the individual participant level or both | Page 7, Lines 142-144 |
| Outcomes | 6a | Completely defined pre-specified primary and secondary outcome measures, including how and when they were assessed | Whether outcome measures pertain to the cluster level, the individual participant level or both | Yes, Page 8, Lines 154 & 164 |
|  | 6b | Any changes to trial outcomes after the trial commenced, with reasons |  | Yes, this is described in the Methods section, Page 11, Lines 222-228. |
| Sample size | 7a | How sample size was determined | Method of calculation, number of clusters(s) (and whether equal or unequal cluster sizes are assumed), cluster size, a coefficient of intracluster correlation (ICC or *k*), and an indication of its uncertainty | Cluster size, other determinants and ICC were calculated and reported in the trial protocol paper. Reference (16) to the trial protocol paper is given in the methods section of the manuscript. See Page 7, Lines 127-129. |
|  | 7b | When applicable, explanation of any interim analyses and stopping guidelines |  | The are also specified in the trial protocol paper (Ref 16). Singh G, Sharma S, Bansal RK et al. A home-based, primary-care model for epilepsy care in India: Basis and design. *Epilepsia Open* 2019; **4**:264-274. See Page 268 |
| Randomisation: | | | |  |
| Sequence generation | 8a | Method used to generate the random allocation sequence |  | Specified in detail in the trial protocol paper (Ref 16) and mention of this made in the current manuscript. Page 7, Line 24 |
|  | 8b | Type of randomisation; details of any restriction (such as blocking and block size) | Details of stratification or matching if used | NA; No stratification or matching was done |
| Allocation concealment mechanism | 9 | Mechanism used to implement the random allocation sequence (such as sequentially numbered containers), describing any steps taken to conceal the sequence until interventions were assigned | Specification that allocation was based on clusters rather than individuals and whether allocation concealment (if any) was at the cluster level, the individual participant level or both | Specified in detail in the trial protocol paper (Ref 16) |
| Implementation | 10 | Who generated the random allocation sequence, who enrolled participants, and who assigned participants to interventions | Replace by 10a, 10b and 10c | Specified in detail in the trial protocol paper (Ref 16) |
|  | 10a |  | Who generated the random allocation sequence, who enrolled clusters, and who assigned clusters to interventions | Specified in detail in the trial protocol paper (Ref 16) |
|  | 10b |  | Mechanism by which individual participants were included in clusters for the purposes of the trial (such as complete enumeration, random sampling) | Yes, Page 7, Lines 121-122 |
|  | 10c |  | From whom consent was sought (representatives of the cluster, or individual cluster members, or both), and whether consent was sought before or after randomisation | Yes, Page 7, Lines 125-126 |
|  |  |  |  |  |
| Blinding | 11a | If done, who was blinded after assignment to interventions (for example, participants, care providers, those assessing outcomes) and how |  | Blinding was at best partial because of the nature of the trial. Nevertheless, we ensured some level of blinding in the assessment as the outcome assessment was done by a study nurse different from the care providers. See Page 7, Lines 135-138 |
|  | 11b | If relevant, description of the similarity of interventions |  | NA |
| Statistical methods | 12a | Statistical methods used to compare groups for primary and secondary outcomes | How clustering was taken into account | Yes, Page 9, Lines 187-189 |
|  | 12b | Methods for additional analyses, such as subgroup analyses and adjusted analyses |  | Adjustment for attrition was done in the multivariate analyses. See Page 9, Lines 182-184; Page 10, Lines 199-200 |
| Results | | | |  |
| Participant flow (a diagram is strongly recommended) | 13a | For each group, the numbers of participants who were randomly assigned, received intended treatment, and were analysed for the primary outcome | For each group, the numbers of clusters that were randomly assigned, received intended treatment, and were analysed for the primary outcome | Yes, We had provided a flow diagram in the trial protocol paper and reproduce this in the supplementary material accompanying the manuscript (Fig. 2s). |
|  | 13b | For each group, losses and exclusions after randomisation, together with reasons | For each group, losses and exclusions for both clusters and individual cluster members | Yes, Page 11, Line 238 to Page 12, Line 242 |
| Recruitment | 14a | Dates defining the periods of recruitment and follow-up |  | Yes, Page 11, Lines 225-230 |
|  | 14b | Why the trial ended or was stopped |  | NA |
| Baseline data | 15 | A table showing baseline demographic and clinical characteristics for each group | Baseline characteristics for the individual and cluster levels as applicable for each group | Yes, Table 1, Pages 22 & 23 |
| Numbers analysed | 16 | For each group, number of participants (denominator) included in each analysis and whether the analysis was by original assigned groups | For each group, number of clusters included in each analysis | Yes, Page 12, Line 249 |
| Outcomes and estimation | 17a | For each primary and secondary outcome, results for each group, and the estimated effect size and its precision (such as 95% confidence interval) | Results at the individual or cluster level as applicable and a coefficient of intracluster correlation (ICC or k) for each primary outcome | NA, the use of a cluster design was mainly from logistic considerations. Although results are presented both clusterwise and individually, we do not think clusters impacted the outcome analysis. |
|  | 17b | For binary outcomes, presentation of both absolute and relative effect sizes is recommended |  | Outcomes are presented as incidence rate ratios and regression coefficients. |
| Ancillary analyses | 18 | Results of any other analyses performed, including subgroup analyses and adjusted analyses, distinguishing pre-specified from exploratory |  | Yes, Page 14, Line 279 to Page 14, Line 313 |
| Harms | 19 | All important harms or unintended effects in each group (for specific guidance see CONSORT for harms^[[3]](#endnote-3)^) |  | Yes, Page 14, Line 315-321 |
| Discussion | | | |  |
| Limitations | 20 | Trial limitations, addressing sources of potential bias, imprecision, and, if relevant, multiplicity of analyses |  | Yes, Our Discussion on trial limitations is pretty extensive; from Oage 15, Line 339 to Page 17, Line 376 |
| Generalisability | 21 | Generalisability (external validity, applicability) of the trial findings | Generalisability to clusters and/or individual participants (as relevant) | Yes, Page 18, Lines 397-406 |
| Interpretation | 22 | Interpretation consistent with results, balancing benefits and harms, and considering other relevant evidence |  | Yes, Page 17, Lines 378-395; Page 18, Lines 408-415 |
| Other information | | |  |  |
| Registration | 23 | Registration number and name of trial registry |  | Yes, Page 11, Lines 221-223 |
| Protocol | 24 | Where the full trial protocol can be accessed, if available |  | Yes, Page 11, Lines 223-224 |
| Funding | 25 | Sources of funding and other support (such as supply of drugs), role of funders |  | Yes, Page 20, Line 423 |

Table s2a: Comparison of baseline characteristics of subjects in the clinic-based arm who completed the trial and those in whom assessments of ASM adherence were prematurely discontinued due to loss to follow-up.

| **Characteristics** | |  | | Statistical significance  (P) |
| --- | --- | --- | --- | --- |
|  |  | Completed trial (n=76) | Prematurely exited trail (n=44) |  |
| Age at entry to trial (years)  Mean ± SD  95% Confidence Interval for Mean  Lower -Upper BoundBound  Median (IQR) | | 25±15  22-29  23(14-32) | 26±16  22-31  23(17-35) | 0.619 |
| Duration of epilepsy (years)  Mean ± SD  95% Confidence Interval for Mean  Lower -Upper Bound Bound  Median (IQR) | | 12±10 | 14±10 | 0.366 |
|  |  | 10-14 | 11-17 |  |
|  |  | 9(3-18) | 12(5-21) |  |
| Age of epilepsy onset (years)  Mean ± SD  95% Confidence Interval for Mean  Lower -Upper Bound Bound  Median (IQR) | | 14±13 | 15±15 | 0.927 |
|  |  | 12-17 | 10-19 |  |
|  |  | 12(5-20) | 10(4-23) |  |
| Gender | Female | 28(37%) | 11(25%) | 0.182 |
| Religion | Hindu | 45(59%) | 29(66%) | 0.670 |
|  | others | 1(1%) | 1(2%) |  |
|  | Sikh | 30(39%) | 14(32%) |  |
| Ethnic origin | Interstate migrant | 28(37%) | 19(43%) | 0.493 |
|  | Local | 48(63%) | 25(57%) |  |
| Education | Illiterate | 41(54%) | 20(45%) | 0.370 |
|  | literate | 35(46%) | 24 (55%) |  |
| Occupation | Employed | 27(36%) | 15(34%) | 0.874 |
|  | Unemployed | 49(64%) | 29(66%) |  |
| Family income | <INR18000/month | 73(96%) | 42(95%) | 0.874 |
|  | >INR18000/month | 3(4%) | 2(5%) |  |
| Social class | Lower | 64(84%) | 34(77%) | 0.344 |
|  | Upper | 12(16%) | 10(23%) |  |
| Marital status | Married | 24(32%) | 18(41%) | 0.302 |
|  | Single/Divorced/Separated | 52(68%) | 26(59%) |  |
| Habitat | Rural | 9(12%) | 5(11%) | 0.937 |
|  | Urban | 67(88%) | 39(89%) |  |
| Pre-trial seizure frequency | Annual | 4(5%) | 5(11%) | 0.396 |
|  | Biannual | 14(18%) | 5(11%) |  |
|  | Daily | 4(5%) | 6(14%) |  |
|  | Monthly | 26(34%) | 15(34%) |  |
|  | Sporadic | 20(26%) | 10(23%) |  |
|  | Weekly | 8(11%) | 3(7%) |  |

Note: * - modified according to Kuppuswamy scale for socioeconomic status, version 2015 (Ref. 19); ** in past 2 years; ^#^Mann-Whitney test (as data not normally distributed), ^##^Chi-square test, ^###^ Wilcoxon rank test; Abbreviations - SD - standard deviation; IQR - interquartile range; INR - Indian National Rupees.

Table s2a: Comparison of baseline characteristics of subjects in the home-based arm who completed the trial and those in whom assessments of ASM adherence were prematurely discontinued due to loss to follow-up.

| **Characteristics** | | attrition | | Statistical significance (P) |
| --- | --- | --- | --- | --- |
|  |  | Completed trial (n=97) | Prematurely exited trail (n=23) |  |
| Age at entry to trial (years)  Mean ± SD  95% Confidence Interval for Mean  Lower -Upper Bound Bound  Median (IQR) | | 26±15  23-29  25(15-36) | 30±17  23-37  32(17-42) | 0.257 |
|  |  |  |  |  |
| Duration of epilepsy (years)  Mean ± SD  95% Confidence Interval for Mean  Lower -Upper Bound Bound  Median (IQR) | | 15±12  13-18  12(7-21) | 14±11  9-18  11(6-21) | 0.655 |
|  |  |  |  |  |
| Age of epilepsy onset (years)  Mean ± SD  95% Confidence Interval for Mean  Lower -Upper Bound Bound  Median (IQR) | | 13±12  10-15  10(3-20) | 19±15  12-25  13(9-27) | 0.057 |
|  |  |  |  |  |
| Gender | Female | 31 (32%) | 9 (39%) | 0.512 |
| Religion | Hindu | 54 (56%) | 11 (48%) | 0.790 |
|  | others | 4 (4%) | 1 (4%) |  |
|  | Sikh | 39 (40%) | 11 (48%) |  |
| Ethnic origin | Interstate migrant | 33 (34%) | 10 (43%) | 0.395 |
|  | Local | 64 (66%) | 13 (57%) |  |
| Education | Illiterate | 33 (34%) | 10 (43%) | 0.395 |
|  | literate | 64 (66%) | 13 (57%) |  |
| Occupation | Employed | 37 (38%) | 9 (39%) | 0.930 |
|  | Unemployed | 60 (62%) | 14 (61%) |  |
| Family income | <INR18000/month | 92 (95%) | 23 (100%) | 0.266 |
|  | >INR18000/month | 5 (5%) | 0 (0%) |  |
| Social class | Lower | 73 (75%) | 23 (100%) | 0.008 |
|  | Upper | 24 (25%) | 0 (0%) |  |
| Marital status | Married | 36 (37%) | 11 (48%) | 0.344 |
|  | Single/Divorced/Seperated | 61 (63%) | 12 (52%) |  |
| Habitat | Rural | 22 (23%) | 1 (4%) | 0.073 |
|  | Urban | 75 (77%) | 22 (96%) |  |
| Pre-trial seizure frequency | Annual | 6 (6%) | 1 (4%) | 0.393 |
|  | Biannual | 11 (11%) | 4 (17%) |  |
|  | Daily | 9 (9%) | 0 (0%) |  |
|  | Monthly | 28 (29%) | 4 (17%) |  |
|  | Sporadic | 34 (35%) | 10 (43%) |  |
|  | Weekly | 9 (9%) | 4 (17%) |  |

Note: * - modified according to Kuppuswamy scale for socioeconomic status, version 2015 (Ref. 19); ** in past 2 years; ^#^Mann-Whitney test (as data not normally distributed), ^##^Chi-square test, ^###^ Wilcoxon rank test; Abbreviations - SD - standard deviation; IQR - interquartile range; INR - Indian National Rupees.

Table s3: Comparison of baseline characteristics of subjects who completed the trial and those in whom assessments of monthly seizure aggregates were prematurely discontinued due to loss to follow-up.

| Characteristics | Category | Completed trail (n=194) | Prematurely exited trail (n=46) | Statistical significance  (P) |
| --- | --- | --- | --- | --- |
| Age at entry to trial (years)  Mean ± SD  95% Confidence Interval  Median (IQR) | | 25 ± 15  23-27  23 (14-34) | 30 ± 16  25.4-35  28 (18-41) | 0.059^#^ |
| Age of onset (years)  Mean ± SD  95% Confidence Interval  Median (IQR) | | 13 ± 12  12-15.0  11 (4-20) | 18 ± 16  13-23  13 (6-27) | 0.100^#^ |
| Duration of epilepsy (years)  Mean ± SD  95% Confidence Interval  Median (IQR) | | 14 ± 11  15-Dec  11 (5-19) | 14.8 ± 11  11.6-18  12 (6-22) | 0.319^#^ |
| Arm | Home | 105 (54%) | 15 (33%) | 0.009^##^ |
|  | Clinic | 89 (46%) | 31 (67%) |  |
| Gender | Female | 64 (33%) | 15 (33%) | 0.961^##^ |
| Religion | Hindu | 112 (58%) | 27 (59%) | 0.339^##^ |
|  | Sikh | 77 (40%) | 17 (37%) |  |
|  | Others | 5 (1%) | 2 (0%) |  |
| Ethnic origin | Punjabi | 126 (65%) | 24 (52%) | 0.108^##^ |
|  | Migrant | 68 (35%) | 22 (48%) |  |
| Education* | Literate | 102 (53%) | 29 (63%) | 0.2^##^ |
|  | Illiterate | 92 (47%) | 17 (37%) |  |
| Occupation* | Employed  Unemployed | 70 (36%) | 21 (46%) | 0.229^##^ |
|  |  | 124 (64%) | 25 (54%) |  |
| Family income* | < 18000 | 186 (96%) | 44 (96%) | 0.945^##^ |
|  | > 18000 | 8 (4%) | 2 (4%) |  |
| Social class* | Lower | 156 (80%) | 38 (83%) | 0.734^##^ |
|  | Upper | 38 (20%) | 8 (17%) |  |
| Marital status | Married | 69 (36%) | 20 (43%) | 0.318^##^ |
|  | Single/Divorced/Separated | 125 (64%) | 26 (57%) |  |
| Habitat | Rural | 31 (16%) | 6 (13%) | 0.62^##^ |
|  | Urban | 163 (84%) | 40 (87%) |  |
| Pre-trial seizure frequency** | Daily | 16 (8%) | 3 (7%) | 0.804^###^ |
|  | Weekly | 17 (9%) | 7 (15%) |  |
|  | Monthly | 59 (30%) | 14 (30%) |  |
|  | Biannual | 27(14%) | 7(15%) |  |
|  | Annual | 14(7%) | 2(4%) |  |
|  | Sporadic | 61(31%) | 13(28%) |  |

Note: * - modified according to Kuppuswamy scale for socioeconomic status, version 2015 (Ref. 19); ** in past 2 years; ^#^Mann-Whitney test (as data not normally distributed), ^##^Chi-square test, ^###^ Wilcoxon rank test; Abbreviations - SD - standard deviation; IQR - interquartile range; INR - Indian National Rupees.

Table S4. Comparison of monthly cluster-wise proportions of good adherence to ASMs and their effect estimates in the two arms of the trial.

| Month | Unadjusted means of proportion (95%CI) | | Adjusted means of proportion (95%CI)* | | Effect estimates | | | | | |
| --- | --- | --- | --- | --- | --- | --- | --- | --- | --- | --- |
|  | Clinic- based | Home -based | Clinic- based | Home -based | Unadjusted | | | Adjusted | | |
|  |  |  |  |  | Difference** | Ratio^#^ | p-value | Difference** | Ratio^#^ | p-value |
| 1 | 0.67 (0.11 to 1.24) | 0.62 (0.06 to 1.19) | 0.64 (0.08 to 1.21) | 0.66 (0.97 to 1.23) | -0.05 | 0.93 | 0.901 | 0.02 | 1.03 | 0.963 |
| 2 | 0.61 (0.04 to 1.17) | 0.73 (0.17 to 1.30) | 0.67 (0.10 to 1.23) | 0.71 (0.15 to 1.28) | 0.12 | 1.20 | 0.760 | 0.04 | 1.06 | 0.914 |
| 3 | 0.59 (0.03 to 1.16) | 0.69 (0.13 to 1.26) | 0.72 (0.15 to 1.28) | 0.72 (0.15 to 1.28) | 0.10 | 1.17 | 0.809 | 0.00 | 1.00 | 0.999 |
| 4 | 0.66 (0.09 to 1.22) | 0.71 (0.15 to 1.28) | 0.75 (0.18 to 1.32) | 0.72 (0.16 to 1.29) | 0.05 | 1.08 | 0.895 | -0.02 | 0.97 | 0.930 |
| 5 | 0.68 (0.11 to 1.24) | 0.75 (0.18 to 1.31) | 0.81 (0.24 to 1.37) | 0.77 (0.20 to 1.33) | 0.07 | 1.10 | 0.861 | -0.04 | 0.95 | 0.919 |
| 6 | 0.67 (0.11 to 1.24) | 0.78 (0.22 to 1.35) | 0.83 (0.26 to 1.39) | 0.81 (0.24 to 1.37) | 0.11 | 1.16 | 0.786 | -0.02 | 0.98 | 0.967 |
| 7 | 0.75 (0.19 to 1.32) | 0.85 (0.28 to 1.41) | 0.98 (0.42 to 1.55) | 0.89 (0.32 to 1.46) | 0.10 | 1.13 | 0.816 | -0.09 | 0.91 | 0.816 |
| 8 | 0.84 (0.27 to 1.40) | 0.89 (0.33 to 1.46) | 1.16 (0.60 to 1.73) | 0.96 (0.39 to 1.53) | 0.05 | 1.06 | 0.892 | -0.20 | 0.83 | 0.622 |
| 9 | 0.82 (0.25 to 1.39) | 0.85 (0.28 to 1.41) | 1.05 (0.48 to 1.62) | 0.93 (0.36 to 1.50) | 0.03 | 1.04 | 0.949 | -0.12 | 0.89 | 0.765 |
| 10 | 0.83 (0.27 to 1.40) | 0.83 (0.27 to 1.40) | 1.03 (0.47 to 1.60) | 0.94 (0.37 to 1.51) | 0.00 | 1.00 | 0.998 | -0.09 | 0.91 | 0.819 |
| 11 | 0.79 (0.23 to 1.36) | 0.89 (0.32 to 1.45) | 1.15 (0.58 to 1.71) | 0.99 (0.42 to 1.55) | 0.10 | 1.13 | 0.820 | -0.16 | 0.86 | 0.634 |
| 12 | 0.80 (0.23 to 1.36) | 0.91 (0.34 to 1.47) | 1.08 (0.52 to 1.65) | 0.91 (0.39 to 1.52) | 0.11 | 1.14 | 0.789 | -0.17 | 0.84 | 0.755 |
| 13 | 0.88 (0.32 to 1.45) | 0.88 (0.31 to 1.44) | 1.27 (0.70 to 1.84) | 1.05 (0.48 to 1.61) | 0.00 | 1.00 | 0.986 | -0.22 | 0.83 | 0.585 |
| 14 | 0.82 (0.26 to 1.39) | 0.98 (0.42 to 1.55) | 1.20 (0.63 to 1.77) | 1.04 (0.48 to 1.61) | 0.16 | 1.20 | 0.697 | -0.16 | 0.87 | 0.697 |
| 15 | 0.83 (0.26 to 1.39) | 0.94 (0.37 to 1.50) | 1.03 (0.46 to 1.59) | 0.97 (0.48 to 1.53) | 0.11 | 1.13 | 0.788 | -0.06 | 0.94 | 0.884 |
| 16 | 0.80 (0.23 to 1.36) | 0.96 (0.39 to 1.52) | 0.95 (0.39 to 1.52) | 0.95 (0.38 to 1.51) | 0.16 | 1.20 | 0.692 | 0.00 | 1.00 | 0.992 |
| 17 | 0.79 (0.23 to 1.36) | 0.93 (0.37 to 1.50) | 1.47 (0.90 to 2.04) | 1.16 (0.60 to 1.73) | 0.14 | 1.18 | 0.731 | -0.31 | 0.79 | 0.455 |
| 18 | 0.91 (0.34 to 1.47) | 0.94 (0.37 to 1.50) | 1.36 (0.79 to 1.92) | 1.15 (0.58 to 1.72) | 0.03 | 1.03 | 0.937 | -0.21 | 0.85 | 0.614 |
| 19 | 0.82 (0.26 to 1.39) | 0.95 (0.39 to 1.52) | 1.00 (0.43 to 1.56) | 0.97 (0.40 to 1.53) | 0.13 | 1.16 | 0.747 | -0.03 | 0.97 | 0.943 |
| 20 | 0.89 (0.32 to 1.45) | 0.81 (0.24 to 1.37) | 0.9 (0.33 to 1.46) | 0.89 (0.33 to 1.46) | -0.08 | 0.91 | 0.843 | -0.01 | 0.99 | 0.991 |
| 21 | 0.83 (0.26 to 1.39) | 0.8 (0.24 to 1.37) | 3.68 (3.11 to 4.25) | 2.00 (1.43 to 2.56) | -0.03 | 0.96 | 0.957 | -1.68 | 0.54 | 0.000 |
| 22 | 0.87 (0.30 to 1.44) | 0.94 (0.37 to 1.50) | 2.81 (2.24 to 3.37) | 1.67 (1.10 to 2.24) | 0.07 | 1.08 | 0.866 | -1.14 | 0.59 | 0.005 |
| 23 | 0.82 (0.25 to 1.39) | 0.91 (0.34 to 1.47) | 1.46 (0.90 to 2.03) | 1.12 (0.55 to 1.68) | 0.09 | 1.11 | 0.829 | -0.34 | 0.77 | 0.396 |
| 24 | 0.85 (0.28 to 1.41) | 0.80 (0.23 to 1.36) | 1.40 (0.83 to 1.96) | 1.07 (0.50 to 1.63) | -0.05 | 0.94 | 0.903 | -0.33 | 0.76 | 0.415 |

*Adjusted for baseline demographic and clinical variables as well as duration of participation in the trial; ** Effect estimate, i.e., difference in means of proportion of good adherence between the two arms, adjusted for baseline demographic and clinical variables as well as duration of participation in the trial = Mean of proportion in home-care – mean of proportion in clinic -care; # ratio of means of proportion of good adherence between the two arms, adjusted for baseline demographic and clinical variables as well as duration of participation in the trial = Mean of proportion in home-care/mean of proportion in clinic-care.

Added note: The different proportions and effect estimates for months 21 and 22 may be attributed to the pandemic lockdown; the missing data was not imputed for the above analysis.

Table s5. Regression coefficients for SRMS scores according to trial arm and months of follow-up in a random effects ordinal logistic regression model.

|  | Regression coefficient (95%CI) | P> IZI |
| --- | --- | --- |
| **Home-care arm*** | -0.293 (-0.719 to 0.132) | 0.18 |
| **Assessment month** | 0.118 (0.100 to 0.136) | <0.0001 |

Note: * - when compared to clinic-care arm.

Table s6. Regression coefficients of serial PIES scores after imputation in the two arms fitted with the time and cluster covariates to a random effects linear regression model.

|  | Regression coefficient (95%CI) | P> IZI |
| --- | --- | --- |
| **Home-care arm*** | -0.293 (-0.719 to 0.132) | 0.18 |
| **Assessment month** | 0.118 (0.100 to 0.136) | <0.0001 |
| **Occupation (unemployed)** | 6.198 (2.265 to 10.132) | 0.002 |
| **Pre-trial seizure frequency (Daily seizures)** | 3.546 (0.275 to 6.818) | 0.034 |
| **Seizure-freedom throughout trial period (Breakthrough seizures)** | 18.391 (9.777 to 27.005) | P=0.0001 |

Note: * - when compared to clinic-care arm. Apart from the trial arm and time, only those covariates, which remained significant in the model are represented in the table. Number of observations: 1,716; Overall model probability > chi2 = 0.00001. Parentheses in columns 1 and 2 (variables) contain the reference category against which comparison was made.

Table s7. Incidence rate ratios of aggregates of all and neurological adverse effects accrued at monthly intervals in the two arms in a two-level random effects Poisson regression model.

|  | Incidence rate ratio (95% CI) | P> IZI |
| --- | --- | --- |
| *All adverse effects* | | |
| **Assessment month** | 0.95 (0.94 to 0.97) | <0.0001 |
| **Home- care arm** | 0.94 (0.77 to 1.15) | 0.572 |
| *CNS adverse effects* | | |
| **Assessment month** | 0.95 (0.94 to 0.96) | <0.0001 |
| **Home- care arm** | 0.90 (0.78 to 1.04) | 0.165 |

Table s8. Sensitivity analysis – 1. Regression coefficients for acceptable adherence spread over the first 18 months (after excluding the lockdown period) in the two arms in a random effects logistic regression model allowing for between cluster variability.

|  | Regression coefficient (95%CI) | P> IZI |
| --- | --- | --- |
| **Home-care arm*** | 0.687 (0.325 to 1.048) | <0.0001 |
| **Assessment month** | 0.075 (0.046 to 0.105) | <0.0001 |

Note: * - when compared to clinic-care arm; Number of observations = 4320 (Observation per group = 18; Prob > chi2 =0.00001.

Table s9. Sensitivity analysis – 2. Regression coefficients for monthly seizure aggregates after imputation spread over the first 18 months (after excluding the lockdown period) in the two arms in a random effects negative binomial regression model allowing for between cluster variability.

|  | Regression coefficient (95%CI) | P> IZI |
| --- | --- | --- |
| **Home-care arm*** | -2.279 (-3.868 to -0.971) | <0.001 |
| **Assessment month** | -0.481 (-0.619 to -0.344) | <0.0001 |
| **Age of onset of epilepsy** | -0.026 (-0.048 to -0.003) | 0.025 |
| **Pre-trial seizure frequency**  **Sporadic seizures (Daily seizures)** | -0.634 (-1.300 to -0.008) | 0.047 |
| **Biannual seizures (Daily seizures)** | -1.190 (-1930 to 0.451) | 0.002 |

Note: * - when compared to clinic-care arm; Number of observations = 4320 (Observation per group = 18; Prob > chi2 =0.00001; ; Only variables that were statistically significant in the model are presented here. ; Number of observations = 4320 (Observation per group = 18).

Training component

The initial training activity took place in the first one month and would be repeated at six monthly intervals. Two levels of training activities were undertaken:

8.1.1Training of study personnel**:** The study personnel sanctioned by the ICMR include one SRF and two field workers. The SRF is a postgraduate (MSc) in Nursing and the two field workers with ANMs/GNMs (Diploma in Nursing) and therefore of the same qualification as ANMs. The study personnel were trained in the following two areas: (1) Basic epilepsy diagnosis and management and (2) Study protocols and procedures. The training took place according the following two phases: (a) Induction training: This took place in the first one month. The study personnel prepared and presented seminars on basic epilepsy topics with a neurologist as the preceptor. A total of 14 topics were discussed during these seminars. The duration of each seminar was one hour and the list of topics is given in Table 1 below. The syllabus and curriculum for the study personnel were based on (1) Guidelines for epilepsy management in India (GEMIND,2008)(12) (b) Follow-up training: A weekly program comprising 3 sessions every month including one seminar on epilepsy, a journal club on a study-related aspect or protocol and study protocol or analysis discussion meeting has been drawn out (Table 2). This schedule will be adhered throughout the study period.

**Box 1:- List of seminar for the study personnel**

| **S. NO.** | **DAY / DATE/ TIME** | **TOPICS** | **PRESENTED BY** | **INSTRUCTORS** |
| --- | --- | --- | --- | --- |
| 1 | Monday  (5-6-2017)  3-4pm | Definition, classification,  Prevalence & incidence of epilepsy | Mrs. Suman | Dr. Gagandeep Singh |
| 2 | Tuesday  (6-6-2017)  3-4pm | Investigation of epilepsy | Mrs. Shivani | Dr. Jatinder Singh Goraya |
| 3 | Wednesday  (7-6-2017)  3-4pm | Drug treatment of epilepsy | Ms. Allina | Dr. Jatinder Singh Goraya |
| 4 | Thursday  (8-6-2017)  3-4pm | Drug Adverse effects of epilepsy | Ms Sukhpreet | Dr. R.K. Bansal |
| 5 | Friday  (9-6-2017)  3-4pm | Seizure, safety driving | Mrs Shivani | Dr Gagandeep Singh |
| 6 | Saturday  (10-6-2017)  3-4pm | Women with epilepsy including pregnancy | Ms Allina | Dr Gagandeep Singh |
| 7 | Monday  (12-6-2017)  3-4pm | First aid in seizures | Ms Sukhpreet | Dr. J.S. Goraya |
| 8 | Tuesday  (13-6-2017)  3-4pm | Children with epilepsy | Mrs Suman | Dr. J.S. Goraya |
| 9 | Wednesday  (14-6-2017)  3-4pm | Surgery in epilepsy | Mrs Suman | Dr. J.S. Goraya |

**Box 2:-list of journal club & Protocol discussions.**

| S. No. | Day/Date | Topic | Presentator |
| --- | --- | --- | --- |
| 1 | Wednesday,  July 19, 2017 | Journal club | Dr. Salil uppal |
| 2 | Friday  July 21, 2017 | Anatomy & Physiology of CNS | Ms Allina |
| 3 | Saturday  July 22, 2017 | Study protocol (PIES) | Discussion |
| 4 | Wednesday,  july26,2017 | Journal club | Dr. Jasloveleen |
| 5 | Friday  july27,2017 | Definition, classification, Etiology and investigation of epilepsy | Ms Sukhpreet |
| 6 | Saturday  july28,2017 | Study protocol(QOiLE) | Discussion |
| 7 | Wednesday,  Aug 2,2017 | Journal club | Ms Suman |
| 8 | Friday  Aug 4,2017 | Medical Treatment and adverse effects of AED_s_ | Ms Allina |
| 9 | Saturday  Aug 5,2017 | Study protocol | Discussion |
| 10 | Wednesday  Aug 9,2017 | Journal club | Dr. Parveen |
| 11 | Friday  Aug 11,2017 | Syncope, Non Epileptic seizure | Ms Sukhpreet |
| 12 | Saturday  Aug 12,2017 | Study protocol | Discussion |
| 13 | Wednesday  Aug 16,2017 | Journal club | Sister Ranjit |
| 14 | Friday  Aug 18,2017 | How to ensure drug adherence? | Ms Allina |
| 15 | Saturday  Aug 19,2017 | Study protocol | Discussion |
| 16 | Wednesday  Aug 23,2017 | Journal club | Dr. Salil uppal |
| 17 | Friday  Aug 25,2017 | Status epilepsy | Ms Sukhpreet |
| 18 | Saturday  Aug 26,2017 | Study protocol | Discussion |
| 19 | Wednesday  Aug 30,2017 | Journal club | Dr. Jasloveleen |
| 20 | Friday  Sept 1^st^ ,2017 | Medical, social and legal issues of epilepsy | Ms Sukhpreet |
| 21 | Saturday  Sept 3^rd^ ,2017 | Study protocol | Discussion |
| 22 | Wednesday  Sept 6,2017 | Journal club | Ms Suman |
| 23 | Friday  Sept 8 ,2017 | Children with epilepsy | Ms Allina |
| 24 | Saturday  Sept 9 ,2017 | Study protocol | Discussion |
| 25 | Wednesday  Sept 13,2017 | Journal club | Dr. Parveen |
| 26 | Friday  Sept 15 ,2017 | Surgery in epilepsy | Ms Sukhpreet |
| 27 | Saturday  Sept 16,2017 | Study protocol | Discussion |
| 28 | Wednesday  Sept 20,2017 | Journal club | Sister Ranjit |
| 29 | Friday  Sept22,2017 | Care of epileptic patients at home | Ms Allina |
| 30 | Saturday  Sept 23 ,2017 | Study protocol | Discussion |
| 31 | Wednesday  Sept 27,2017 | Journal club | Dr. Salil uppal |
| 32 | Friday  Sept29 ,2017 | Complications of epilepsy | Ms Sukhpreet |
| 33 | Saturday  Sept30 ,2017 | Study protocol | Discussion |
| 34 | Wednesday  Oct 4,2017 | Journal club | Dr. Jasloveleen |
| 35 | Friday  Oct 6,2017 | Ketogenic diet | Ms Allina |
| 36 | Saturday  Oct 7,2017 | Screening protocol | Discussion |
| 37 | Wednesday  Oct 11,2017 | Journal club | Ms suman |
| 38 | Friday  Oct 13,2017 | Women with epilepsy | Ms Sukhpreet |
| 39 | Saturday  Oct14,2017 | Study protocol | Discussion |
| 40 | Wednesday  Oct 18,2017 | Journal club | Dr. Parveen |
| 41 | Friday  Oct 20,2017 | Driving & safety advices for epilepsy | Ms Allina |
| 42 | Saturday  Oct 21,2017 | Study protocol | Discussion |
| 43 | Wednesday  Oct 25,2017 | Journal club | Sister Ranjit |
| 44 | Friday  Oct 27,2017 | Health education for epileptic patients | Ms Sukhpreet |
| 45 | Saturday  Oct 28,2017 | Screening protocol | Discussion |

8.1.2 Training simulation of District Health Personnel: Although the District Health Personnel were not directly involved in the project, a training simulation exercise was undertaken in as much as training of health care providers at all levels in basic epilepsy management is critical to the success of any national epilepsy care program. The objective of this simulation exercise was to demonstrate the feasibility and usefulness of training health care providers in basic epilepsy management. The training simulation exercise took place during the first month and included one CME each (conducted in 2 batches) for the following three levels of health care providers:

- - - 1. Medical Officers in charge of primary health care (total: 34; actually attended)
      2. ANMs (total: 123; actually attended)
      3. Accredited Social Health activists (ASHA) workers (total: 290; actually attended)


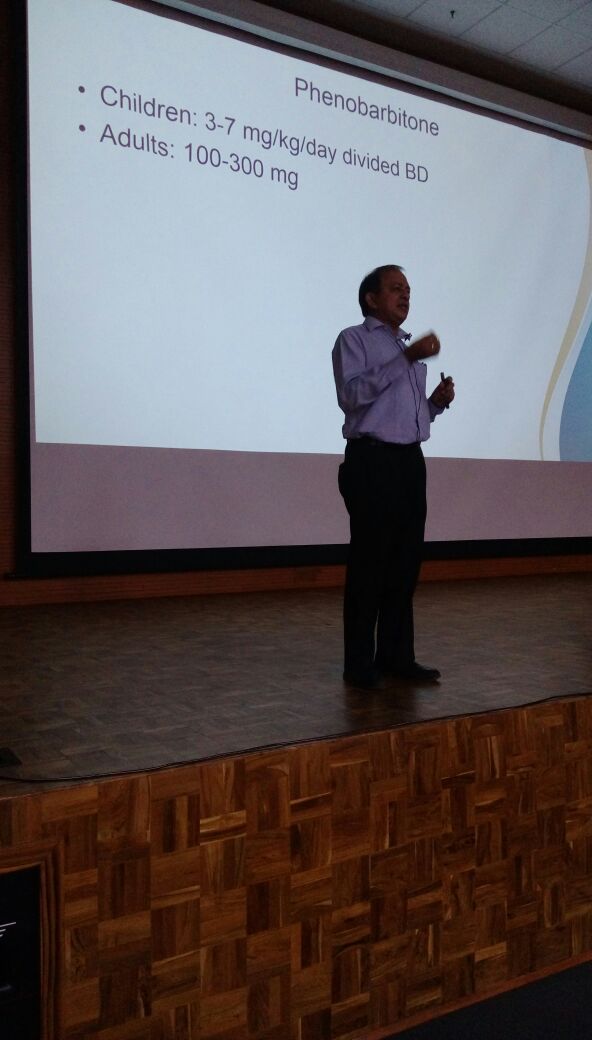


**Fig 4 : Study personnel delivered a lecture on antiepileptic drugs (23-6-2017)**


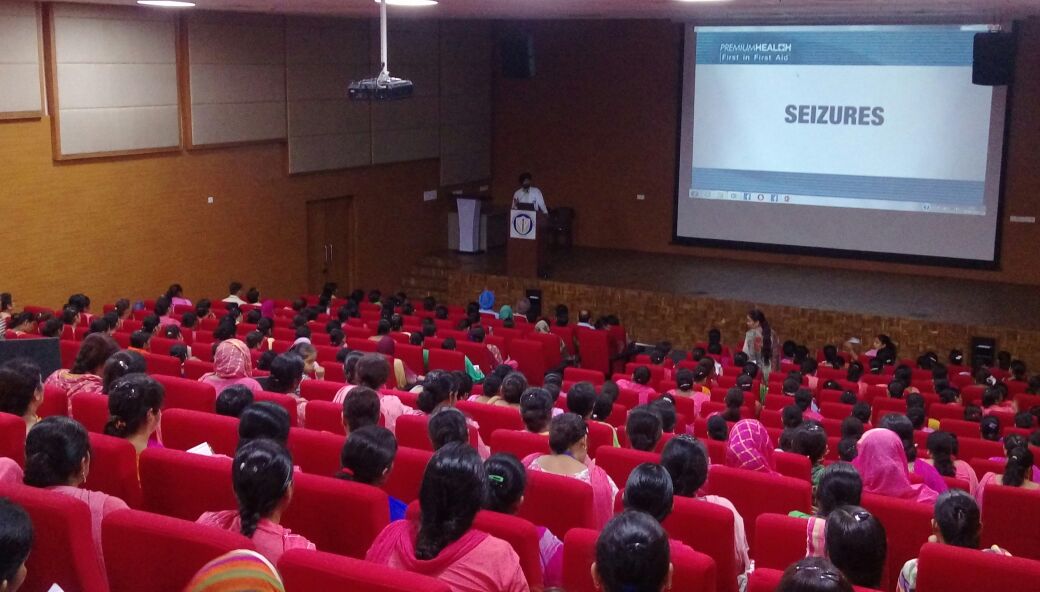


**Fig 5: View of audience attended Epilepsy CME for Accredited Social Health Activist Organized by CIFE study group (30-6-2017)**

Each CME was last for 4 hours and a sample of the curriculum is shown in table. The following was the schedule for the Medical Officer and ANM and ASHA workshop:

**Box 3. Schedule of Scientific Program for Medical Officer’s CME (16-6-2017)**

| **Chair Person & Moderator** – **Dr. Anurag Chaudhary** | | | | |
| --- | --- | --- | --- | --- |
| S. No. | Day / Date | Presentation | Topic | Moderator / Time |
| 1 | Friday,  16- 6-2017  10am -1pm | Medical  officers | Pre. Test | Dr. Rupinder (10 Min). |
|  |  |  | Introduction | Dr. Gagandeep Singh  (15 Min.) |
|  |  |  | Definition & classification of epilepsy | Dr. Jatinder Singh Goraya  (20 Min) |
|  |  |  | Common drugs/adverse effects | Dr. Rajinder Kumar Bansal  (20 Min) |
|  |  |  | Women with epilepsy | Dr. Monika Singla  (20 Min) |
|  |  |  | First aid in epilepsy  &  Driving/safety in epilepsy | Dr. Barinder Singh Paul  (20 Min) |
|  |  |  | Project protocol | Dr. Sarit Sharma  (20 min) |
|  |  |  | Question and answer | Dr. Anurag Chaudhary  &  All speakers (20 min) |
|  |  |  | Post test | Dr. Rupinder  (10 MIN) |
|  |  |  |  | **LUNCH** |

**Box 4: Schedule of Scientific Program for ANM’s CME (23-6-2017)**

| **Chair Person & Moderator** – **Dr. Anurag Chaudhary** | | | | |
| --- | --- | --- | --- | --- |
| S No. | Day / Date | Presentation | Topic | Moderator / Time |
| 1 | Friday  (23-6-2017)  10am -1pm | ANM Batch 1 | Pre. Test | Dr. Rupinder (10Min). |
|  |  |  | Introduction | Dr. Gagandeep Singh (15 Min.) |
|  |  |  | Definition & classification of epilepsy | Dr. Jatinder Singh Goraya  (20 Min) |
|  |  |  | Common drugs/adverse effects | Dr. Rajinder Kumar Bansal  (20 Min) |
|  |  |  | Women with epilepsy | Dr. Monika Singla (20 Min) |
|  |  |  | First aid in epilepsy  &  Driving/safety in epilepsy | Dr. Barinder Singh Paul  (20 Min) |
|  |  |  | Project protocol | Dr. Sarit Sharma (20 min) |
|  |  |  | Post test | Dr. Rupinder (10 min) |
|  |  |  |  | **LUNCH** |
| 2 | Friday  (23-6-2017)  1Pm -4.30pm | ANM Batch for -2 | Pre. Test | Dr. Rupinder (10 Min) |
|  |  |  | Introduction | Dr. Gagandeep Singh  (15 Min.) |
|  |  |  | Definition & classification of epilepsy | Dr. Barinder Singh Paul (20 Min) |
|  |  |  | Common drugs/adverse effects | Dr. Rajinder Kumar Bansal (20 Min) |
|  |  |  | Women with epilepsy  &  First aid in epilepsy | Dr. Barinder Singh Paul  (20 Min) |
|  |  |  | Driving/safety in epilepsy | Dr. Monika Singla (20 Min) |
|  |  |  | Project protocol | Dr. Sarit Sharma (20 min) |
|  |  |  | Post test | Dr. Rupinder (10 Min) |

**Box 5: Schedule of Scientific Program for ASHA WorkersCME (30-6-2017)**

| **Chair Person & Moderator** – **Dr. Anurag Chaudhary** | | | | |
| --- | --- | --- | --- | --- |
| S. No. | DAY / DATE | Presentation | Topic | Moderator / Time |
| 1 | Friday  (30-6-2017)  10am -1pm | ASHA Workers | Pre. Test | Dr. Rupinder (10Min). |
|  |  |  | Introduction | Dr. Gagandeep Singh  (15 Min.) |
|  |  |  | Definition & classification of epilepsy | Dr. Jatinder Singh Goraya  (20 Min) |
|  |  |  | Common drugs/adverse effects | Dr. Rajinder Kumar Bansal  (20 Min) |
|  |  |  | Women with epilepsy | Dr. Monika Singla  (20 Min) |
|  |  |  | First aid in epilepsy  &  Driving/safety in epilepsy | Dr. Barinder Singh Paul  (20 Min) |
|  |  |  | Project protocol | Dr. Sarit Sharma  (20 min) |
|  |  |  | Post test | Dr. Rupinder  (10 MIN) |
|  |  |  |  | **LUNCH** |

8.2 Evaluation of the impact of the training workshops

A pre- and post-test questionnaires were administered to the delegates in order to determine their baseline knowledge about epilepsy and attitudes towards epilepsy and the usefulness (if any) of the educational intervention. The same questionnaire but with a different sequence of items would be administered after three months in order to evaluate the long-term benefits of the educational intervention. It is proposed to repeat the training CME once every six months in order to provide continuous updating as well as education of newly recruited health care workers.

**Model specification and equations for cluster and multivariate analyses**

1. **Univariate analysis after accounting for cluster design**
2. Proportion with good adherence in a given month

E (d_ij_) = m_ij_ π_ij_

where, d = number of participants with good adherence in a given cluster; m = number of individuals in the cluster; and π = proportion of participants with good adherence in the cluster; *i* = individual; *j* = cluster

&

π_ij =_ α + β_i_ +μ_ij_

where, α = expected chances of good adherence in a randomly selected participant in a randomly selected cluster in the absence of intervention (clinic care); β = effect of intervention; and μ = between cluster variability in each treatment arm

1. Monthly seizure aggregates

E (d_ij_) = λ_ij_ θ_ij_

where, λ = number of seizures/month for an *i*th participant in a *j*th cluster; θ = person-months of follow-up; and d = number of seizures in a cluster; *i* = individual; *j* = cluster

&

λ_ij =_ α + β_i_ +μ_i_

where, α = expected number of seizures/month in a randomly selected participant in a randomly selected cluster in the absence of intervention (home care); β = effect of intervention; and μ = between cluster variability in each treatment arm

1. **Multivariate analyses**
2. Attrition

θ_ik_ = α + β_i_ + Σγ_i_ Z_ik_

where θ = expected probability of exiting trial in an *i*^th^ participant in the *k*^th^ arm; α = expected probability of exiting trial in a randomly selected participant in a randomly selected cluster in the absence of intervention (clinic care); β = effect of intervention; γ_i_ represents the effect of covariates z_1_, z_2_....

1. Random effects, Poisson regression for good adherence in a repeated measures design

θ_ijkt_ = α + β_i_ + Σγ_i_ Z_ijkl_ + ζ_t_ + ψ_ijk_ +μ_ij_

where θ = chances of good adherence in an *i*^th^ participant in *j*^th^ cluster in *k*^th^ arm at *t*^th^ month; α = expected chances of good adherence in a randomly selected participant in a randomly selected cluster in the absence of intervention (clinic care); β = effect of intervention; γ_i_ represents the effect of covariates z_1_, z_2_...; ζ= time variable; ψ = within individual correlation over time; and μ = between cluster variability in each treatment arm.

1. Random effects zero inflated negative binomial regression for monthly seizure aggregates

θ_ijkt_ = α + β_i_ + Σγ_i_ Z_ijkl_ + ζ_t_ + ψ_ijk_ +μ_ij_

where θ = expected number of seizures in an *i*^th^ participant in *j*^th^ cluster in *k*^th^ arm at *t*^th^ month; α = expected numbers of seizures in a randomly selected participant in a randomly selected cluster in the absence of intervention (clinic care); β = effect of intervention; γ_i_ represents the effect of covariates z_1_, z_2_...; ζ= time variable; ψ = within individual correlation over time; and μ = between cluster variability in each treatment arm.

1. Hopewell S, Clarke M, Moher D, Wager E, Middleton P, Altman DG, et al. CONSORT for reporting randomised trials in journal and conference abstracts. *Lancet* 2008, 371:281-283 [↑](#endnote-ref-1)
2. Hopewell S, Clarke M, Moher D, Wager E, Middleton P, Altman DG at al (2008) CONSORT for reporting randomized controlled trials in journal and conference abstracts: explanation and elaboration. *PLoS Med* 5(1): e20 [↑](#endnote-ref-2)
3. Ioannidis JP, Evans SJ, Gotzsche PC, O'Neill RT, Altman DG, Schulz K, Moher D. Better reporting of harms in randomized trials: an extension of the CONSORT statement. *Ann Intern Med* 2004; 141(10):781-788. [↑](#endnote-ref-3)
